# HiCdat: a fast and easy-to-use Hi-C data analysis tool

**DOI:** 10.1186/s12859-015-0678-x

**Published:** 2015-09-03

**Authors:** Marc W. Schmid, Stefan Grob, Ueli Grossniklaus

**Affiliations:** Institute of Plant Biology, University of Zurich, Zollikerstrasse 107, Zürich, 8008 Switzerland; Zurich-Basel Plant Science Center, Universitätstrasse 2, Zürich, 8092 Switzerland

**Keywords:** Chromosome Conformation Capture (3C), Nuclear architecture, Hi-C, Data analysis, Sample comparison, Structural domains, Correlation to (epi-)genome

## Abstract

**Background:**

The study of nuclear architecture using Chromosome Conformation Capture (3C) technologies is a novel frontier in biology. With further reduction in sequencing costs, the potential of Hi-C in describing nuclear architecture as a phenotype is only about to unfold. To use Hi-C for phenotypic comparisons among different cell types, conditions, or genetic backgrounds, Hi-C data processing needs to be more accessible to biologists.

**Results:**

HiCdat provides a simple graphical user interface for data pre-processing and a collection of higher-level data analysis tools implemented in R. Data pre-processing also supports a wide range of additional data types required for in-depth analysis of the Hi-C data (e.g. RNA-Seq, ChIP-Seq, and BS-Seq).

**Conclusions:**

HiCdat is easy-to-use and provides solutions starting from aligned reads up to in-depth analyses. Importantly, HiCdat is focussed on the analysis of larger structural features of chromosomes, their correlation to genomic and epigenomic features, and on comparative studies. It uses simple input and output formats and can therefore easily be integrated into existing workflows or combined with alternative tools.

**Electronic supplementary material:**

The online version of this article (doi:10.1186/s12859-015-0678-x) contains supplementary material, which is available to authorized users.

## Background

The development of Chromosome Conformation Capture (3C) techniques and their high throughput derivatives (e.g., 4C and Hi-C) has enabled the analysis of nuclear architecture (i.e. chromatin organization) at an unprecedented resolution [1]. Hi-C data analysis comprises a large variety of approaches, including point-to-point looping interactions (e.g., promoter-enhancer interactions), three-dimensional modeling of chromatin [2], identification of structural domains (e.g., topologically associated domains, TADs [3]), or comparison of different genetic backgrounds (e.g., wild-type *versus* mutant tissues [4–6]).

The large number of reads produced by Hi-C experiments (e.g., around 200–300 mio aligned read-pairs per sample in [3]) requires efficient tools for processing, filtering, and simplification of the data to best match the demands of the downstream analyses. Several open-source tools are available, each with its own scope and requirements. HiCUP [7] performs mapping and quality control on Hi-C data but no downstream analysis. Sushi [8] and HiTC [9] provide data visualization functionality, but no pre-processing or statistical analysis of Hi-C data. HiCseg specifically focusses on identification of domains in Hi-C data [10]. ChromoR [11] offers data pre-processing and sample comparison, but does not support the analysis of additional genomic and epigenomic features. HiCpipe [12] implements a computationally very intense normalization method, which does not perform better than the parametric approach in HiCNorm [13] (normalization method). HOMER [14] and hiclib [15] offer a large variety of functionalities, including pre-processing and higher-level data analysis. However, these tools may be inaccessible to users with limited programming experience: HOMER requires some command-line skills and only generates plain-text output, which needs to be further processed by the user; hiclib requires familiarity with Python. The latter is less well known among molecular biologists and geneticists who are likely more familiar with R. Alternatively, HiBrowse offers many functionalities in an easy-to-use web-interface [16], which, however, constrains the users by forcing them to adhere to the available procedures and the requirement of uploading their data to a web server.

Envisioning nuclear architecture (i.e. chromatin organization) as an ordinary phenotype of an organism or a specific tissue type (e.g. like the transcriptome), comparative Hi-C experiments may soon be of very broad interest, raising the need for data analysis tools that are not only well-accessible to bioinformaticians. We therefore developed HiCdat. It includes a fast and easy-to-use GUI tool for Hi-C data pre-processing and an R [17] package, which implements all data analysis approaches employed in [5].

## Implementation

HiCdat was developed with a focus on speed, user-friendliness, and flexibility in terms of file formats. The GUI tool for data pre-processing serves to convert large-scale genomic and epigenomic data into simple tables, which can be efficiently loaded and processed within R. The R-package provides a collection of functions, which allow higher-level data analysis (e.g., as in [5]) with only a few lines of code. Data formats are kept as simple as possible to ensure that the user can easily integrate HiCdat into a pre-existing workflow or combine it with other tools.

## Results and discussion

**Fig. 1:**
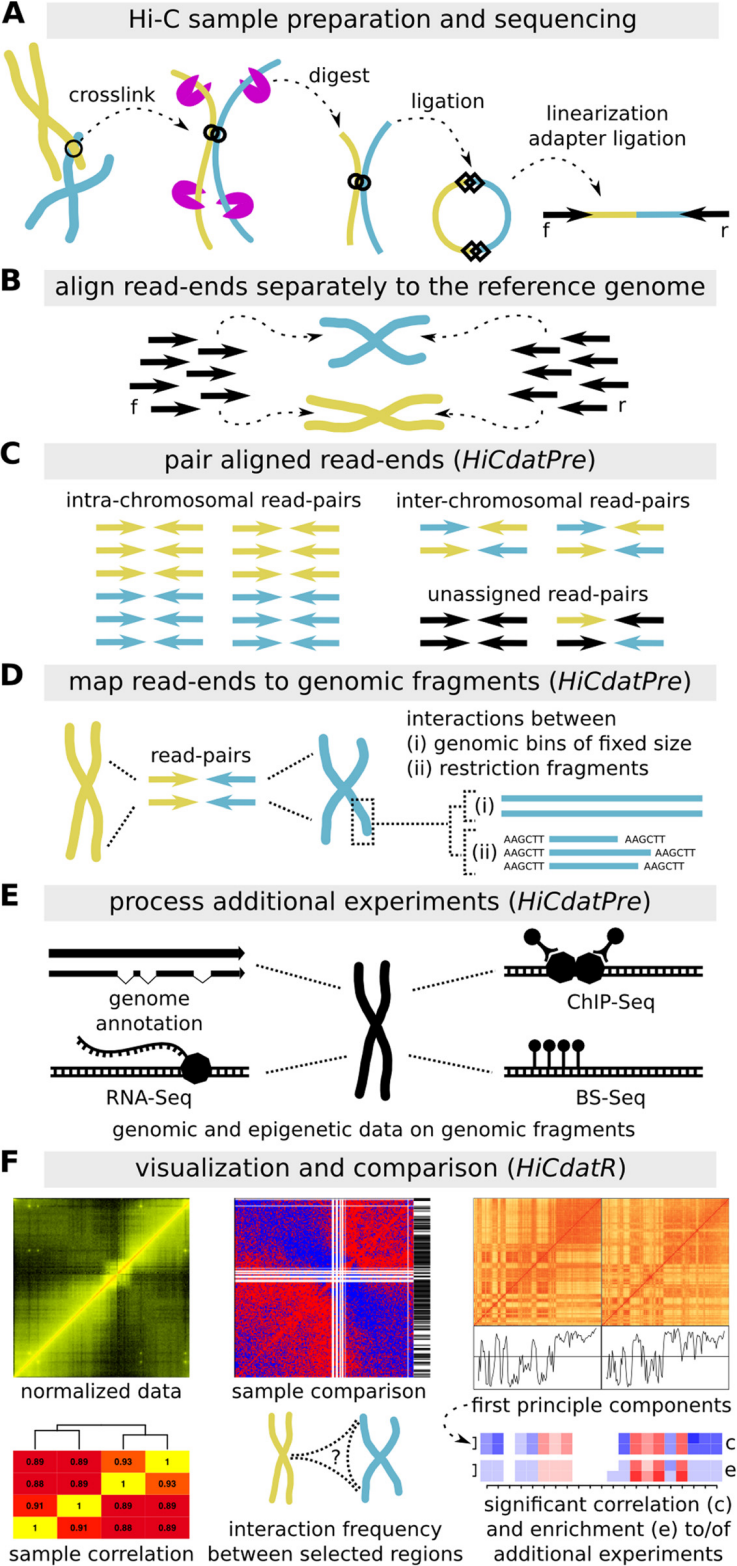
Schematic HiCdat workflow. (**a**-**b**) After sequencing and initial quality checks have been performed, the read-ends (f: forward, r: reverse) are aligned separately to a reference genome. (**c**-**d**) After pairing the separately aligned read-ends, each end is mapped to genomic fragments, which are either genomic bins with a fixed size or restriction fragments with variable size. (**e**) Genomic fragments can be associated with various data types to test for correlation and enrichment of Hi-C data with genomic and epigenomic features. (**f**) Finally, the data can be conveniently analyzed in R using HiCdatR

HiCdat is divided into two parts (Fig. 1): (i) a GUI tool for data pre-processing (termed *HiCdatPre*) and (ii) an R-package for higher-level data analysis (termed *HiCdatR*).

### Data pre-processing with *HiCdat*

*HiCdat* takes as input two alignment files (forward and reverse reads, hereafter termed read-ends) in BAM format (Binary Alignment/Map), a reference genome, and various data types from additional experiments (e.g., genome annotation, RNA-Seq, ChIP-Seq, BS-Seq data). There are five automated steps during data pre-processing: (i) pairing aligned reads, (ii) creating fragments, (iii) mapping of read-ends to fragments, (iv) processing data from additional experiments, and (v) creating organism-specific R-code.

#### Pairing aligned reads

The read-ends are first aligned seperately to the reference genome using, for example, Subread [18]. Uniquely aligning read-ends are then paired based on their common read name to create read-paris (around 12.6 million read-ends per minute^1^).

#### Creating fragments

Hi-C data analysis can either be carried out on restriction fragments or genomic bins with fixed size. Both types of fragments can be created by supplying the reference genome sequence and one or more restriction enzymes or a fixed bin size.

#### Mapping read-ends to fragments

To calculate the interaction frequency between two fragments, the read-pairs are first mapped to the fragments’ coordinates and then summarized as number of interactions per fragment pair (around 7.5 million read-pairs per minute^1^). During this procedure, the read-pairs can optionally be filtered using the approach proposed by [19]. Read-pairs with each end aligning at the opposite strand are thereby removed if they are too close to each other. There are two cases: (i) A read-pair where the two ends point towards each other (“inward-pair”), and (ii) a read-pair where the two ends point away from each other (“outward-pair”). Inward-pairs spanning only a short region may be caused by uncut DNA. Outward-pairs spanning only a short region can be a result of self-ligation.

#### Processing data from additional experiments

To analyze the interplay between the Hi-C interactome and genomic/epigenomic features, a large variety of such information can be automatically added to the fragments. In principle there are two fundamentally different types of data: counts and densities. During higher-level data analysis, counts are generally log-transformed, whereas densities are kept as percentages. Likewise, if data are summarized over multiple fragments (e.g. to obtain the annotation for 1 Mb bins directly from the fragment annotation), counts are summed up, whereas densities are averaged. Both data types comprise two sub-types, resulting in four different types of “tracks” which can be processed: (i) genome annotation features (e.g., genes and transposons), (ii) short count features (e.g., RNA-Seq and smallRNA-Seq), (iii) density features (e.g., ChIP-Seq), and (iv) DNA-methylation density (e.g., BS-Seq).

Genome annotation features (GFF/GTF files with multiple feature types per file) can generally be very long and possibly span multiple fragments. The number of elements per fragment is therefore counted as follows: If the feature spans the entire fragment, a value of 1 is added. If the feature only partly overlaps (or is within) the fragment, a value of 0.5 is added. In contrast, short count features (BAM files with one feature type only) are mostly entirely within a fragment and are therefore simply summed up per fragment.

Density of a certain feature (BAM files with one feature type only) is calculated as the number of bases covered by at least one element (e.g. short read) divided by the length of the fragment (times 100 to obtain percentages). Likewise, DNA cytosin-methylation density corresponds to the percentage of methylated C’s per fragment.

#### Creating the organism-specific R-code

Higher-level data analysis requires some organism-specific R-code, which can be obtained by supplying the reference genome sequence and the restriction enzyme(s) used for the Hi-C library preparation.

### Data analysis with *HiCdatR*

In-depth Hi-C data analysis is done in R with *HiCdatR*. The only inputs required are the interaction counts per fragment pair and, optionally, the annotation of the fragments holding the genomic and epigenomic tracks. For most of the functions, it is furthermore possible to supply tables specifying genomic regions of interest (e.g. chromosome arms or pericentromeres as in [5]). The functionalities include (i) data normalizations as proposed by [13, 20, 21], (ii) sample correlation matrices, (iii) data visualization, (iv) sample comparisons, (v) calculation of distance decay exponents, (vi) principle component analysis (PCA) including correlation of the first principle component to genomic and epigenomic features, (vii) test for increased interaction frequencies between genomic regions of interest compared to randomly sampled regions, and (viii) test for enrichment or depletion of genomic and epigenomic features within genomic regions of interest compared to randomly chosen regions.

#### Data normalization

Multiple data normalization strategies have been proposed and implemented in various languages and packages [11–13, 15, 20, 21]. Three of them have been re-implemented in HiCdat: (i) the distance (*intra*chromosomal interactions) and coverage (*inter*-chromosomal interactions) normalization described in [20], (ii) the iterative coverage normalization proposed by [21], and (iii) the more sophisticated but highly efficient normalization using Poisson regression as implemented in HiCNorm [13], which performs similar or better [11, 13] than the procedures from [12, 15].

#### Sample correlation

To visualize the similarities between samples and replicates, HiCdat uses sample correlation matrices. Correlation between two samples is thereby calculated as the average, or median, correlation between all the individual bins of the interaction matrices (i.e. the virtual 4C tracks, see Additional file 1: Figure S1).

#### Data visualization

Hi-C interaction frequencies and differences between multiple samples are visualized as heatmap-like images. Individual samples can either be displayed natively (i.e. with their normalized interaction frequencies, Additional file 2: Figure S2) or in a correlated manner (Additional file 3: Figure S3).

#### Sample comparison

Three different approaches to compare two samples to each other are implemented. In a first approach, the difference of a given fragment pair between the two samples is divided by the average interaction frequency among the two samples resulting in “relative differences” [4] (Additional file 4: Figure S4). Considering that neighboring genomic regions are physically linked to each other, it is likely that they change accordingly. To visualize these domains, the relative differences can be correlated to each other (“correlated differences”, Additional file 5: Figure S5). The disadvantage of these approaches is that they rely on visual inspection of the difference matrices. To estimate the significance of the difference and identify the affected regions, we introduced signed difference matrices (SDMs) [5]. Additionally, they also provide an overall estimate of the extent and significance of the difference between two samples (Additional file 6: Figure S6).

#### Calculation of distance decay exponents (IDEs)

The extent to which interaction frequencies change dependent on the distance to a given point in the genome can be characterized with the interaction decay exponent (IDE). IDEs are calculated as the slope of a linear fit to the average interaction frequencies observed at given distances (both log-transformed, Additional file 7: Figure S7). IDEs were initially used to predict the folding principles of the human genome using two polymer-folding models (the fractal and equilibrium globule module, respectively), which result in distinct values for the expected IDE [20]. Alternatively, they can also be used to describe differences between certain sub-compartments of the genome, or between samples [5].

#### Identification of structural domains using principle component analysis (PCA)

The correlation between the interactomes of different genomic regions can be used to identify larger compartments [20] or structural domains [5]. The approach relies on principal component analysis (PCA) of the distance-normalized and correlated *intra*-chromosomal interactions (Additional file 8: Figure S8). The first principal component (FPC) can then be used to differentiate for example the A and B compartments in *Homo sapiens* [20], or loose and compact structural domains in *Arabidopsis thaliana* [5]. The interplay between the FPC and the epigenomic/genomic landscape can be analyzed with two methods: (i) either by using the built-in cor.test() [17] function to test for significance of correlation between FPC and the density/count of a given feature (Additional file 9: Figure S9), or (ii) by using an approach in which the fragments are split into two groups according to the sign of the FPC (Additional file 10: Figure S10, Additional file 11: Figure S11). Enrichment of a given feature can then be calculated as the ratio of the average density/count in one over the other group, and tested for significance using a two-sided Wilcoxon rank sum test [5]. For the identification of more refined structural domains, as for example topologically associated domains (TADs), HiCdatR provides a simple wrapper around the HiCseg package [10]. Its algorithm relies on two-dimensional segmentation to identify *cis*-interacting regions, and the results were shown to be in good concordance with biologically confirmed regions [10].

#### Testing selected regions for increased interaction frequency and enrichment/depletion of epigenomic/genomic features

Given a set of genomic regions of interest, HiCdat can test for increased interaction frequencies between the regions of interest compared to randomly sampled regions. Considering that the interactome can be strongly influenced by the linear position of a certain region along the chromosome (e.g. close to telomere or centromere), or the chromosome number itself [5, 22], random sampling is performed in a “balanced” fashion: Within each random set, the randomly chosen regions reflect the numbers, as well as the locations, of the regions of interest. The procedure creates an empirical distribution of interaction frequencies between random sets, which can then be used to calculate an empirical *P*-value (one-sided) for the enrichment of interactions between the sets of interest [5]. The same sampling approach can be applied to test for enrichment or depletion of epigenomic or genomic features within a set of genomic regions of interest.

## Conclusions

In short, HiCdat allows rapid Hi-C data analysis as described in [5], requiring only little programming experience. The focus lies on the identification of larger structural features of chromosomes, their interplay with the epigenomic/genomic landscape, and on comparative studies. Input and output is kept as simple as possible to enable easy integration into pre-existing workflows, or the combination of a part of the tool with another tool.

## Availability and requirements

- **Project name:** HiCdat
- **Project home page:**github.com/MWSchmid/HiCdat
- **Operating systems:** Windows (7), MacOSX (> 10.8), Ubuntu-like Linux distributions (all 64 bit)
- **Programming language:** C++ and R
- **Other requirements:** R-packages: randomizeBE, gplots, MASS, HiCseg [10]
- **License:** GNU GPL v3
- **Any restrictions to use by non-academics:** None

## Endnote

^1^ Run-times were measured on a 64 bit Kubuntu running on an Intel Core i7 930@2.8 GHz with 24 Gb RAM and a 7’200 rpm Samsung HDD using Hi-C data from mouse embryonic stem cell (GSM862720, GSM862721) and cortex (GSM862750, GSM862751) samples from [3] (NCBI37 assembly, and 1 Mb bins for the higher-level data analysis, and 823’377 *Hind*III restriction fragments for mapping to fragments).

## Additional files

Additional file 1
**Figure S1.** Correlation between five samples of *Arabidopsis thaliana* seedlings [4, 5] aligned with either Bowtie [23], Bowtie 2 [24], or Subread [18], and processed with either HiCdat or hiclib [15] using a resolution of 100 kb. (PNG 601 kb)

Additional file 2
**Figure S2.** Visualization of Hi-C interaction frequencies in a pooled wild-type sample of *A. thaliana* [4, 5] (100 kb bins). (PNG 3328 kb)

Additional file 3
**Figure S3.** Visualization of distance-normalized and correlated Hi-C interaction frequencies in a pooled wild-type sample of *A. thaliana* [4, 5] (100 kb bins). (PNG 3102 kb)

Additional file 4
**Figure S4.** Enrichment (blue) and depletion (red) of interaction frequencies in the wild-type compared to the *crowded nuclei4* (*crwn4*) mutant sample of *A. thaliana* [5] (100 kb bins). (PNG 6354 kb)

Additional file 5
**Figure S5.** Correlation of differences between the wild-type and the *crwn4* mutant samples of *A. thaliana* [5] (100 kb bins). (PNG 2877 kb)

Additional file 6
**Figure S6.** Visualization of the difference between the wild-type and *crwn4* mutant samples of *A. thaliana*, [5] using the signed difference matrix (100 kb bins). (PNG 1689 kb)

Additional file 7
**Figure S7.** Distance-dependent decay of interaction frequencies along entire chromosomes in a pooled wild-type sample of *A. thaliana* [4, 5] (100 kb bins). (PNG 872 kb)

Additional file 8
**Figure S8.** Visualization of distance-normalized and correlated Hi-C interaction frequencies (top), the resulting first principle component (mid), and the distribution of the correlation values (bottom). Data shown for the right arms of chromosomes 1, 4, and 5 from a pooled wild-type sample of *A. thaliana* [4, 5] (100 kb bins). (PNG 565 kb)

Additional file 9
**Figure S9.** Significant correlation (blue: positive, red: negative) of the first principle component with various genomic and epigenomic features. Data shown for the right arms of chromosomes 1, 4, and 5 from a pooled wild-type sample of *A. thaliana* [4, 5] (100 kb bins). Additional data from www.arabidopsis.org and [25–30]. (PNG 603 KB)

Additional file 10
**Figure S10.** Significant enrichment (blue) and depletion (red) of genomic and epigenomic features in regions with positive Eigenvalues compared to regions with negative Eigenvalues. Data shown for the right arms of chromosomes 1, 4, and 5 from a pooled wild-type sample of *A. thaliana* [4, 5] (100 kb bins). Additional data from www.arabidopsis.org and [25–30]. (PNG 602 kb)

Additional file 11
**Figure S11.** Distribution of epigenomic and genomic features in the structural domains with either positive (blue) or negative (red) Eigenvalues. Data from www.arabidopsis.org and [25–30]. (PNG 915 kb)

## Abbreviations

GFF: General feature format
GTF: Gene transfer format
BAM: Binary alignment map
IDE: Interaction decay exponent
PCA: Principle component analysis
FPC: First principle component
