# Supplementary figures and images for: HiCdat: a fast and easy-to-use Hi-C data analysis tool
Source: BMC Bioinformatics. 2015 Sep 3;16(1):277. doi: 10.1186/s12859-015-0678-x (PMC4559209; doi:10.1186/s12859-015-0678-x)

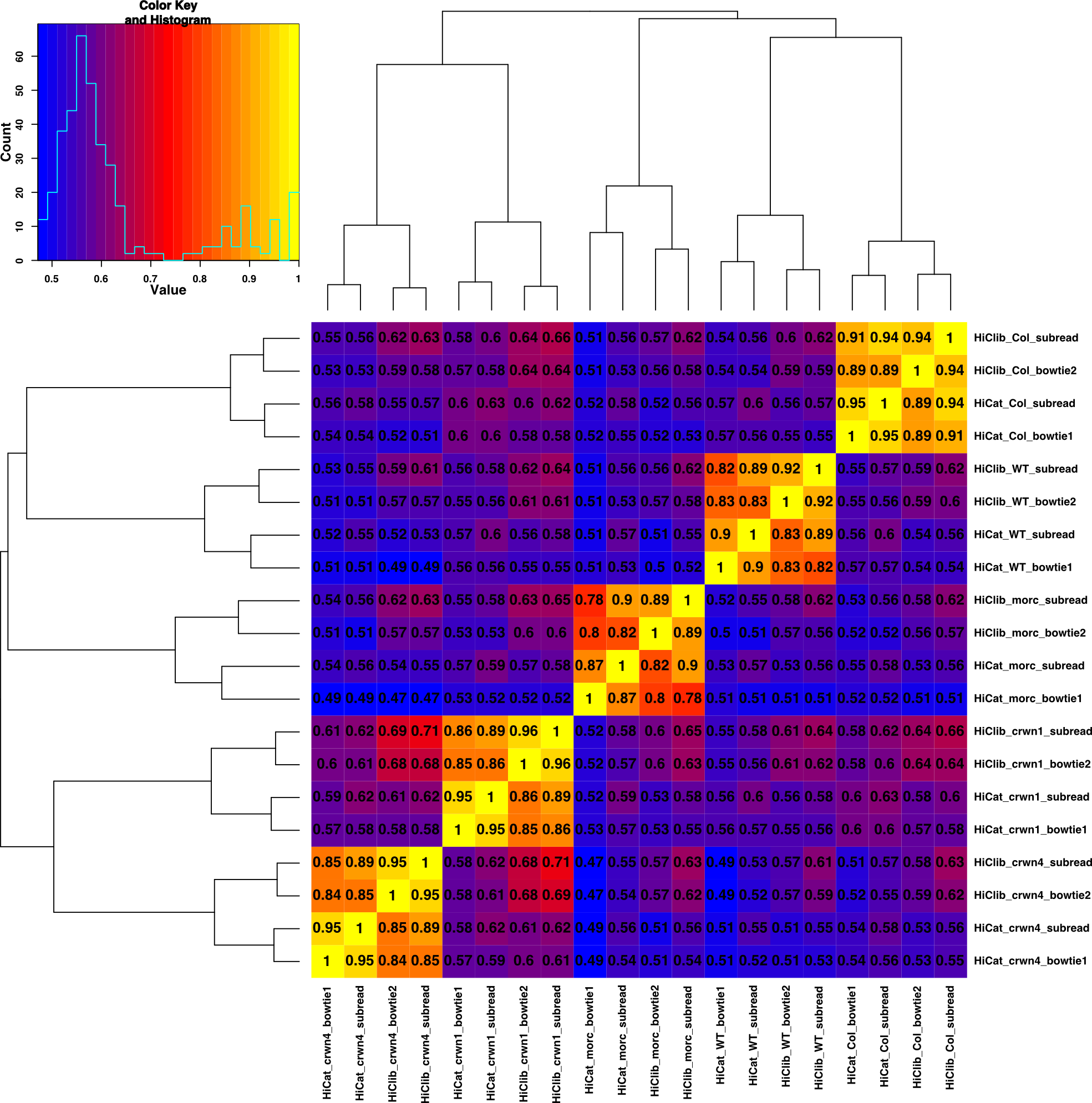

Supplement: Additional file 1 — Figure S1. Correlation between five samples of Arabidopsis thaliana seedlings [4, 5] aligned with either Bowtie [23], Bowtie 2 [24], or Subread [18], and processed with either HiCdat or hiclib [15] using a resolution of 100 kb. (PNG 601 kb) [file 12859_2015_678_MOESM1_ESM.png]

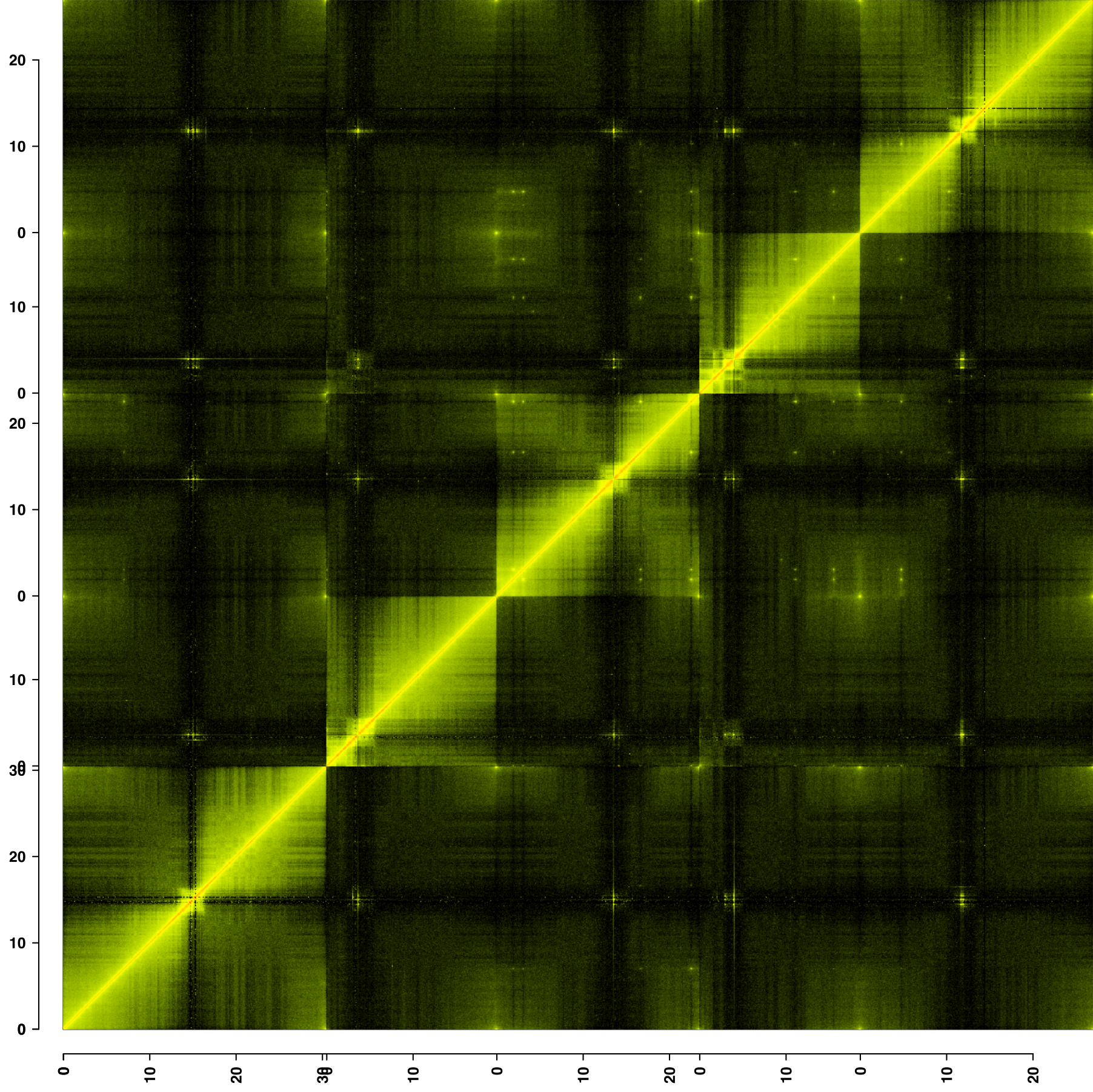

Supplement: Additional file 2 — Figure S2. Visualization of Hi-C interaction frequencies in a pooled wild-type sample of A. thaliana [4, 5] (100 kb bins). (PNG 3328 kb) [file 12859_2015_678_MOESM2_ESM.png]

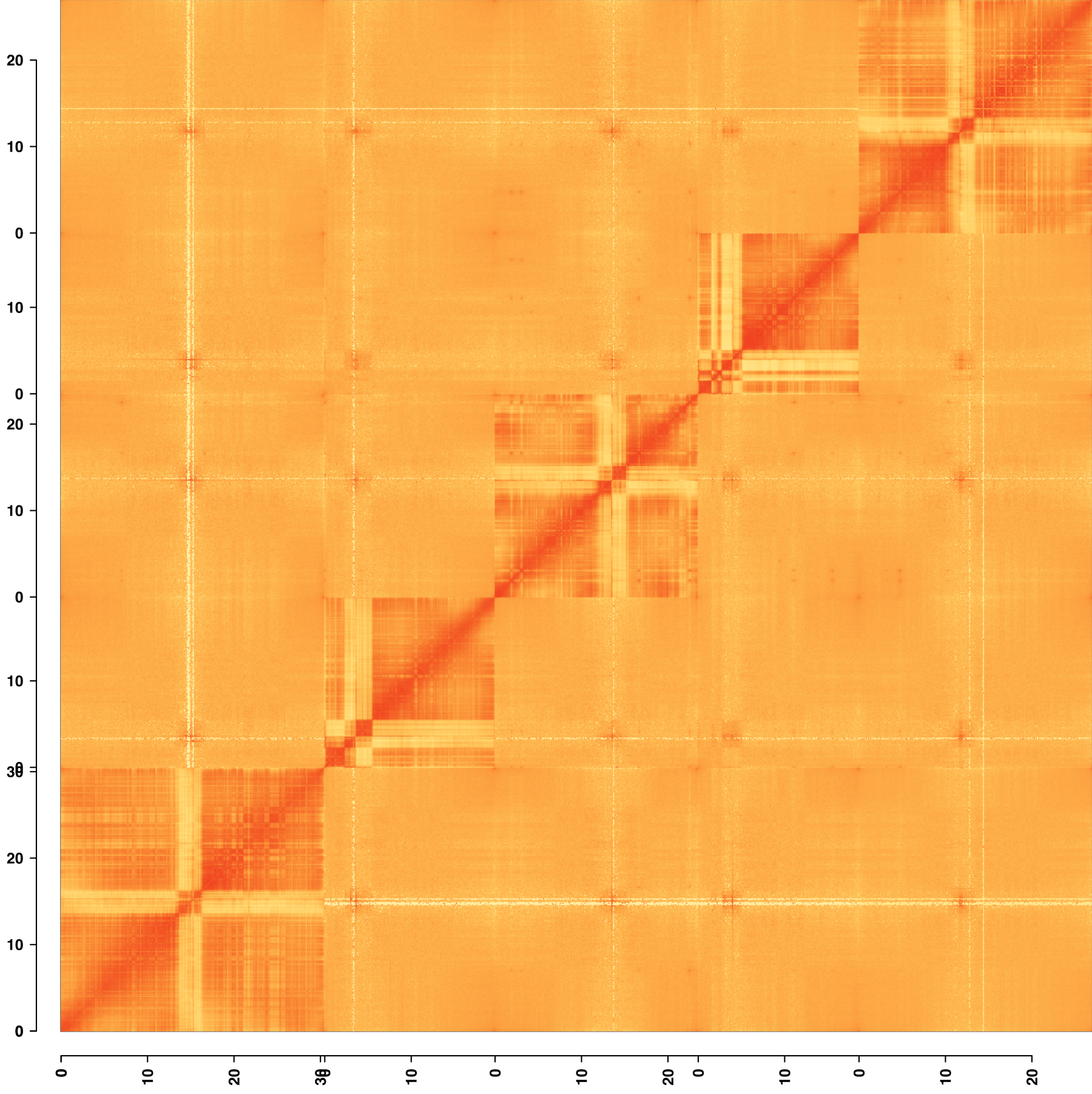

Supplement: Additional file 3 — Figure S3. Visualization of distance-normalized and correlated Hi-C interaction frequencies in a pooled wild-type sample of A. thaliana [4, 5] (100 kb bins). (PNG 3102 kb) [file 12859_2015_678_MOESM3_ESM.png]

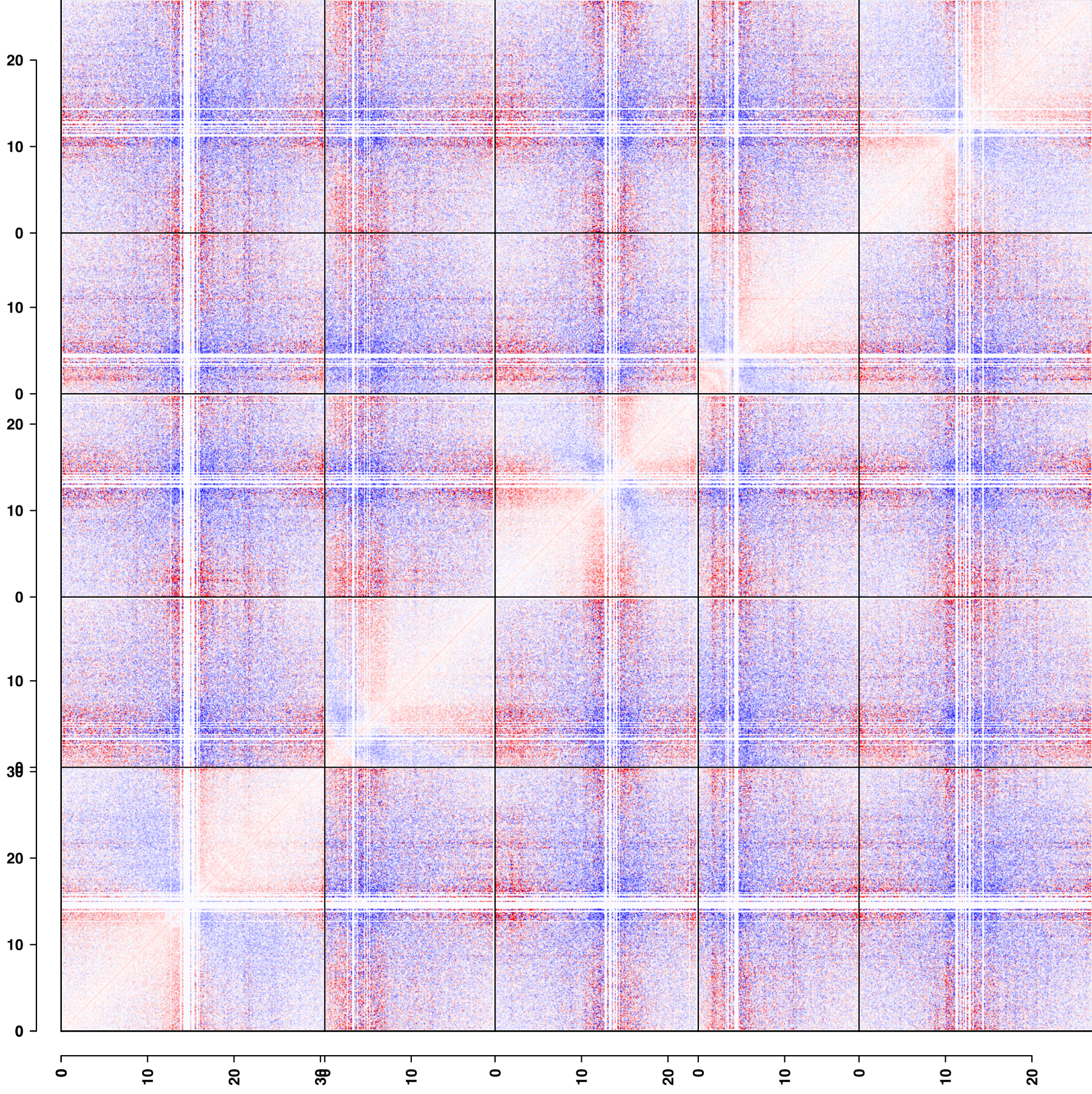

Supplement: Additional file 4 — Figure S4. Enrichment (blue) and depletion (red) of interaction frequencies in the wild-type compared to the crowded nuclei4 (crwn4) mutant sample of A. thaliana [5] (100 kb bins). (PNG 6354 kb) [file 12859_2015_678_MOESM4_ESM.png]

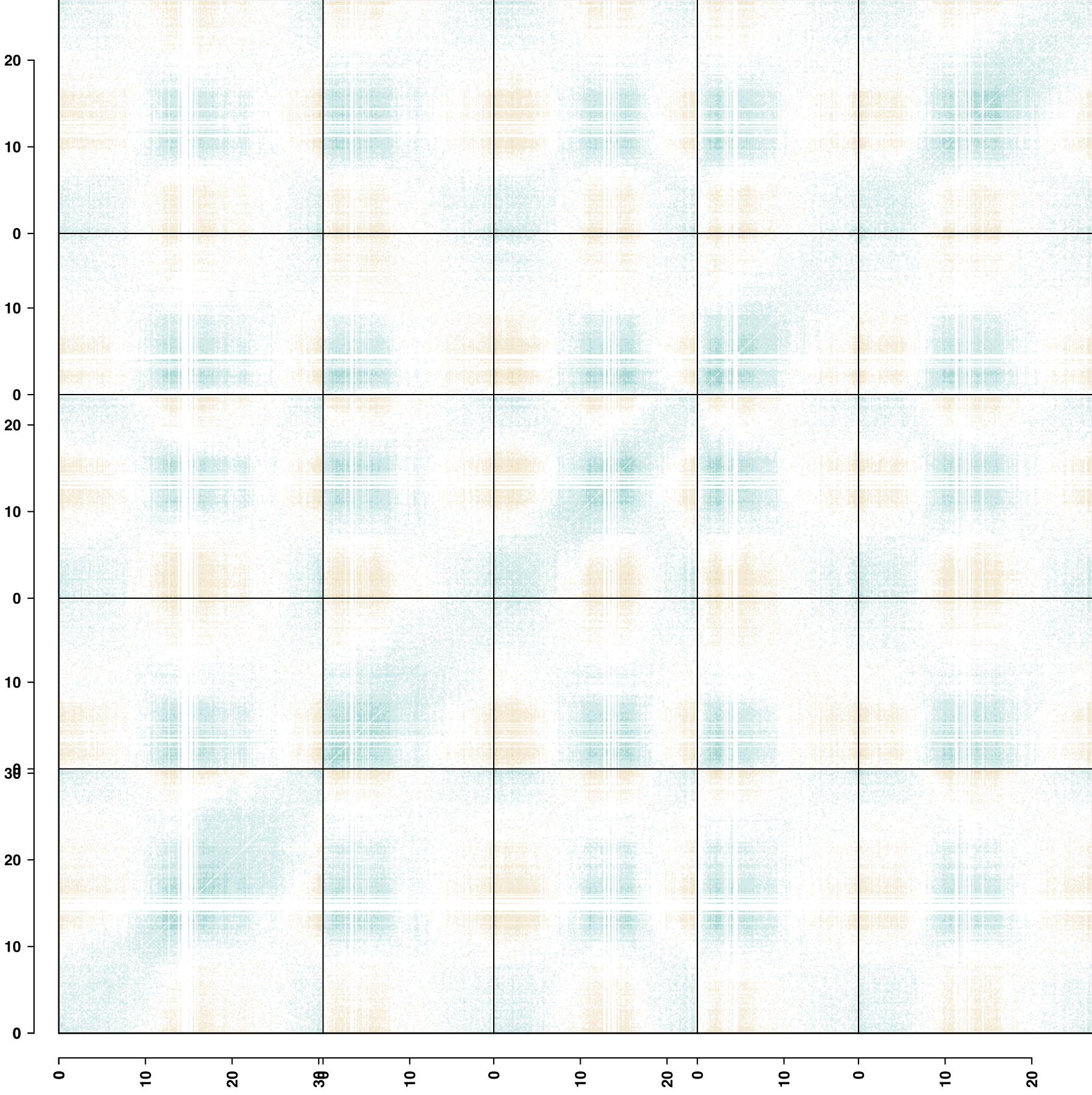

Supplement: Additional file 5 — Figure S5. Correlation of differences between the wild-type and the crwn4 mutant samples of A. thaliana [5] (100 kb bins). (PNG 2877 kb) [file 12859_2015_678_MOESM5_ESM.png]

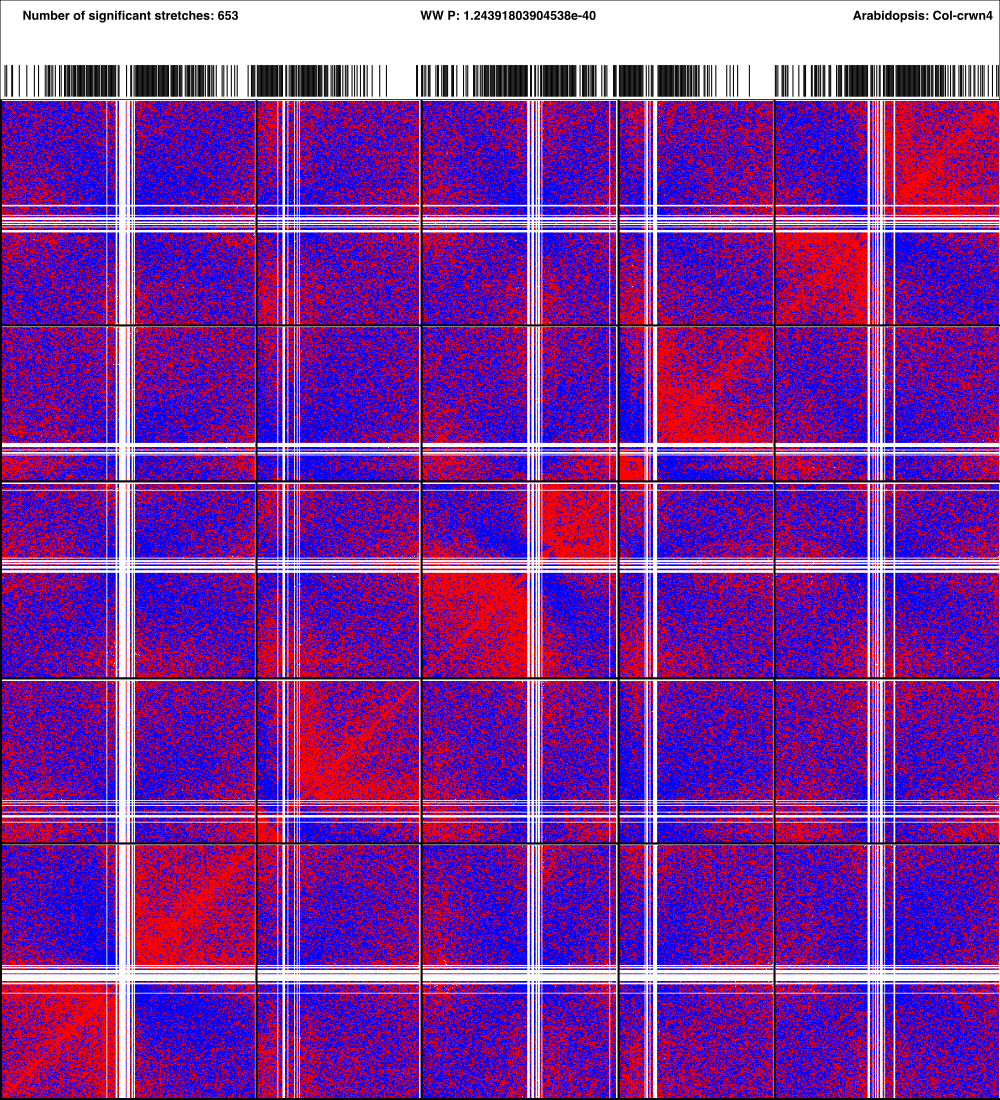

Supplement: Additional file 6 — Figure S6. Visualization of the difference between the wild-type and crwn4 mutant samples of A. thaliana, [5] using the signed difference matrix (100 kb bins). (PNG 1689 kb) [file 12859_2015_678_MOESM6_ESM.png]

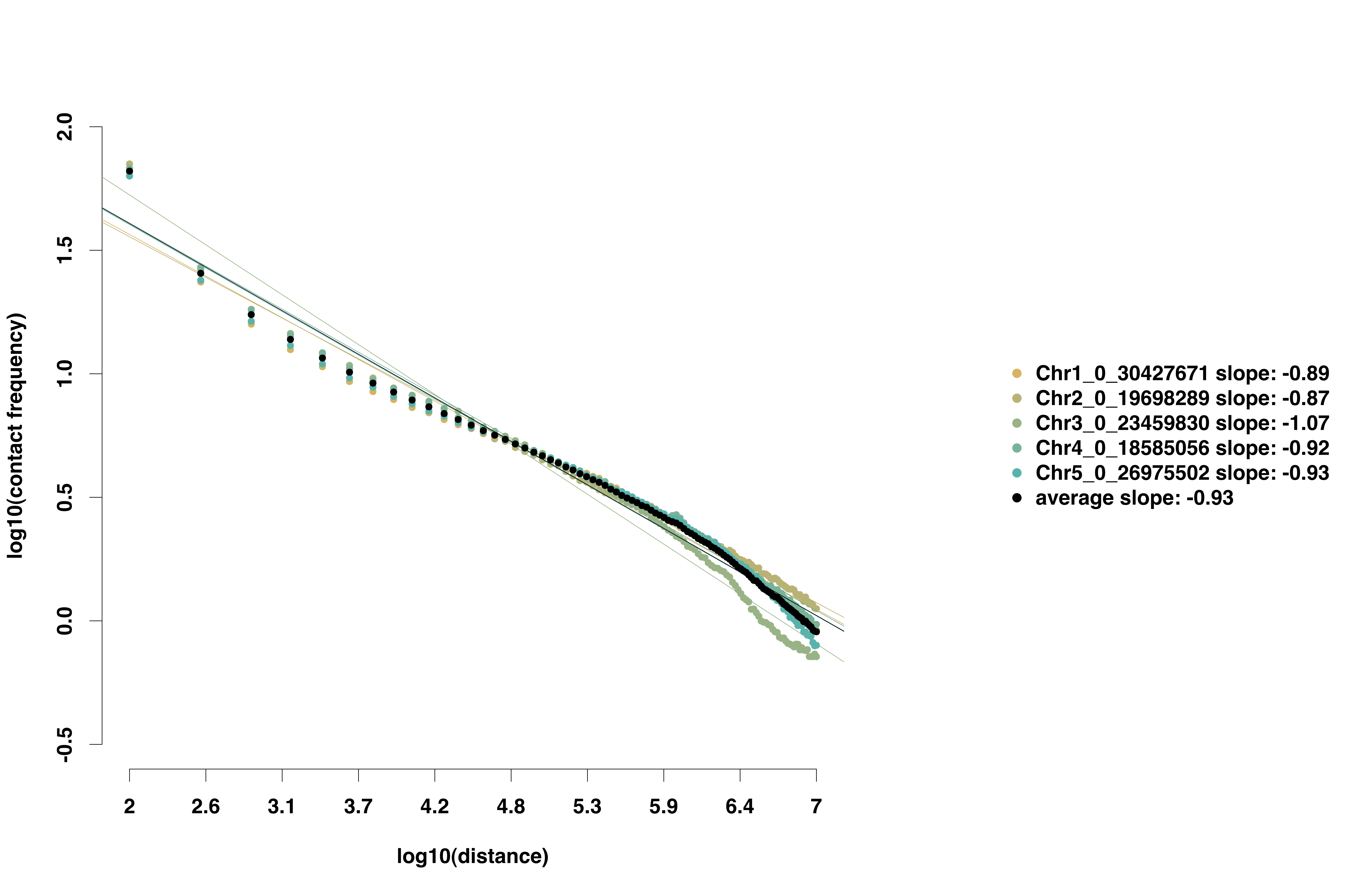

Supplement: Additional file 7 — Figure S7. Distance-dependent decay of interaction frequencies along entire chromosomes in a pooled wild-type sample of A. thaliana [4, 5] (100 kb bins). (PNG 872 kb) [file 12859_2015_678_MOESM7_ESM.png]

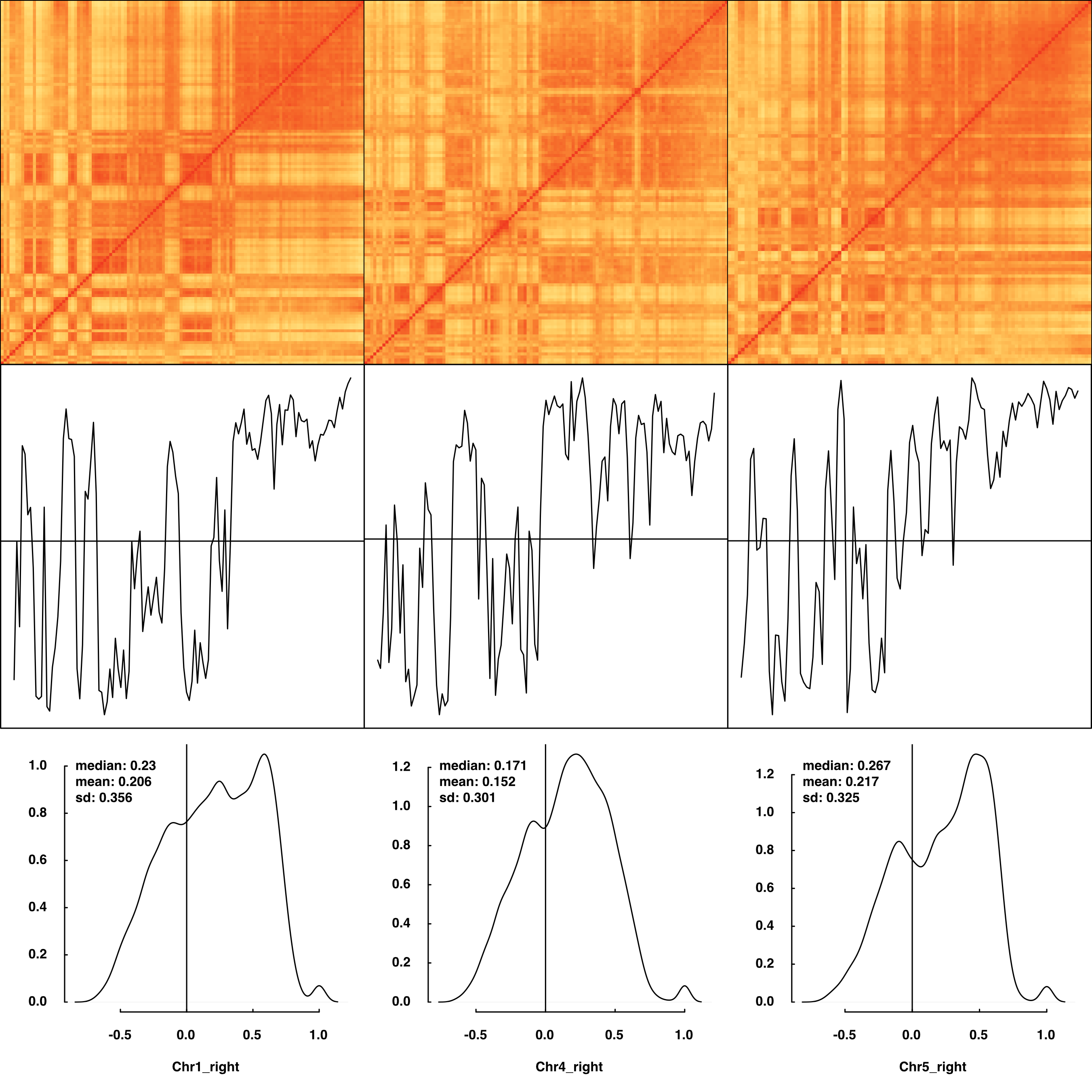

Supplement: Additional file 8 — Figure S8. Visualization of distance-normalized and correlated Hi-C interaction frequencies (top), the resulting first principle component (mid), and the distribution of the correlation values (bottom). Data shown for the right arms of chromosomes 1, 4, and 5 from a pooled wild-type sample of A. thaliana [4, 5] (100 kb bins). (PNG 565 kb) [file 12859_2015_678_MOESM8_ESM.png]

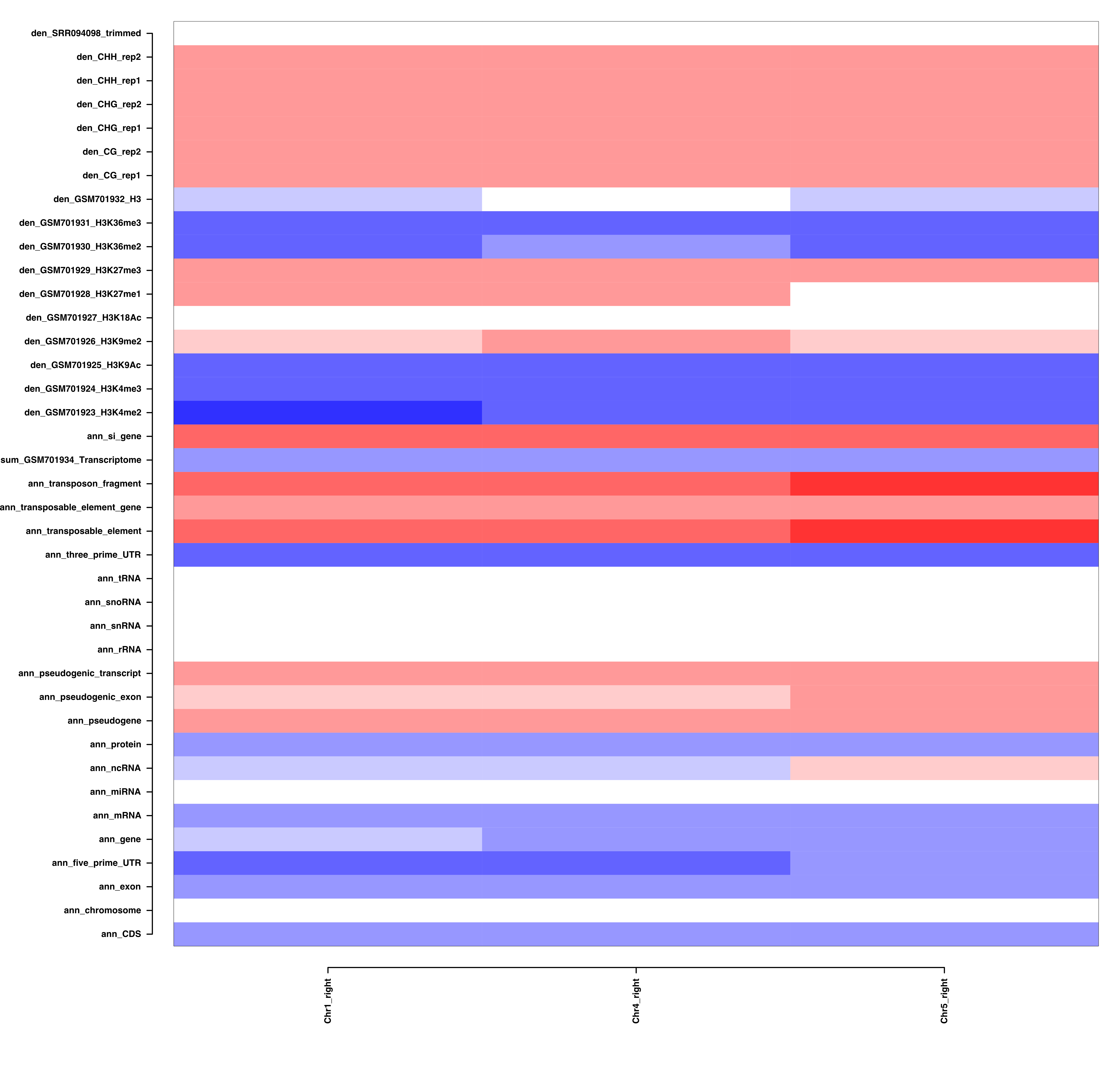

Supplement: Additional file 9 — Figure S9. Significant correlation (blue: positive, red: negative) of the first principle component with various genomic and epigenomic features. Data shown for the right arms of chromosomes 1, 4, and 5 from a pooled wild-type sample of A. thaliana [4, 5] (100 kb bins). Additional data from www.arabidopsis.org and [25–30]. (PNG 603 KB) [file 12859_2015_678_MOESM9_ESM.png]

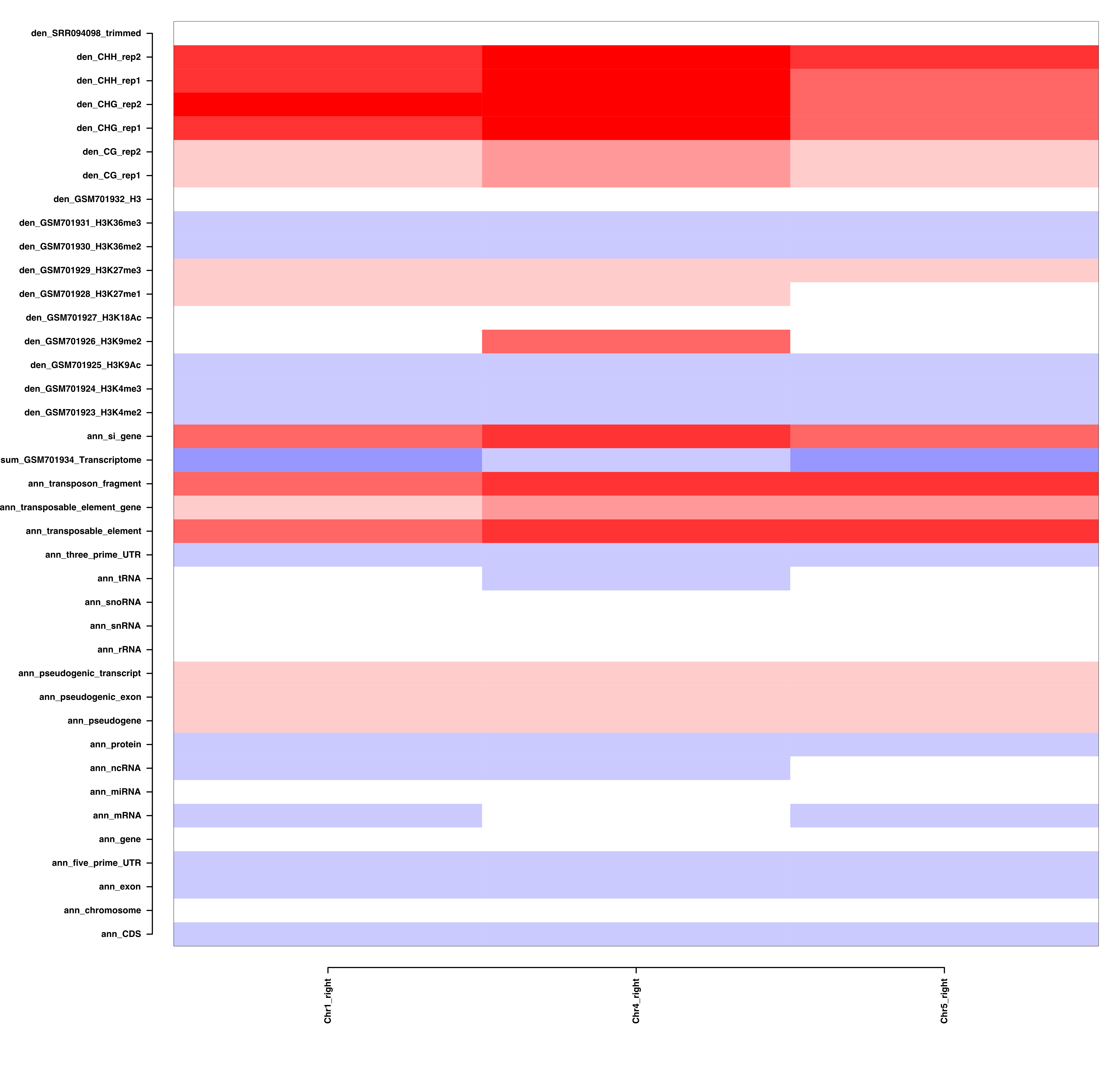

Supplement: Additional file 10 — Figure S10. Significant enrichment (blue) and depletion (red) of genomic and epigenomic features in regions with positive Eigenvalues compared to regions with negative Eigenvalues. Data shown for the right arms of chromosomes 1, 4, and 5 from a pooled wild-type sample of A. thaliana [4, 5] (100 kb bins). Additional data from www.arabidopsis.org and [25–30]. (PNG 602 kb) [file 12859_2015_678_MOESM10_ESM.png]

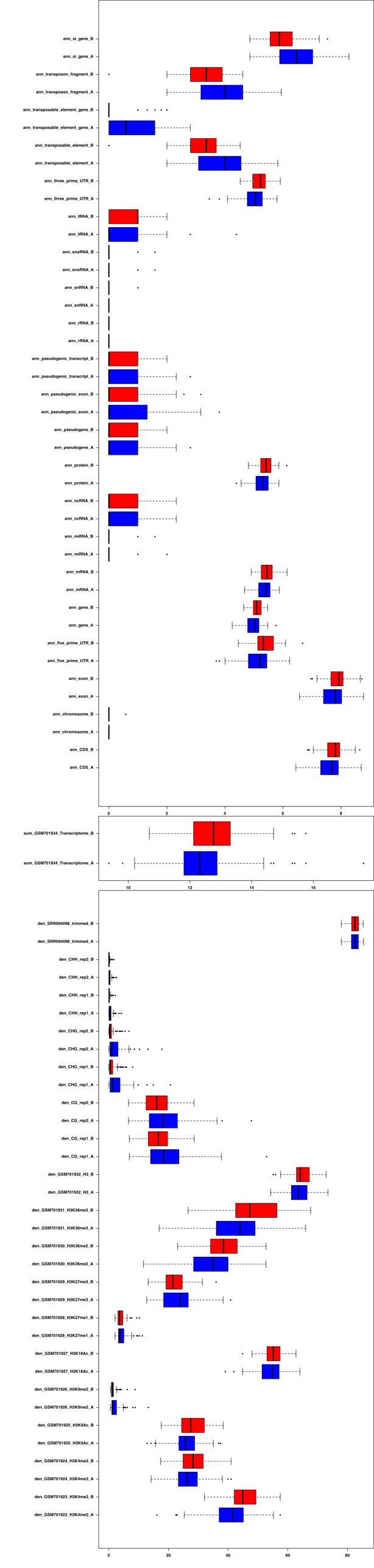

Supplement: Additional file 11 — Figure S11. Distribution of epigenomic and genomic features in the structural domains with either positive (blue) or negative (red) Eigenvalues. Data from www.arabidopsis.org and [25–30]. (PNG 915 kb) [file 12859_2015_678_MOESM11_ESM.png]
